# Evaluation of a random access analyser: BM/Hitachi 911

**DOI:** 10.1155/S1463924695000332

**Published:** 1995

**Authors:** Taweesook Kanluan, Surapon Tangvarasittichai, Orathai Tangvarasittichai

**Affiliations:** Department of Clinical Chemistry Faculty of Medical Technology Mahidol University Siriraj Hospital Bangkok 10700 Thailand

## Abstract

The performance of Boehringer Mannheim's BM/Hitachi 911 was evaluated for three months. The mean coeffcient of variation (CV) of the within-run and between-run imprecision of the 16 analytes were less than 1.16% (range 0.47-2.38%) and 1.35% (range 0.62-2.93,%), respectively. A linearity study for the various assays covered clinically important levels. No relevant drift was observed during an eight-hour assay nor was any sample-related carry-over detected. In all cases, the regression analyses (slopes) of the results obtainedfrom BM/Hitachi 911 and 717 were between the extreme values of 0.94 and 1.05. During the three months of operation, no major problem was encountered. The BM/Hitachi 911 was found to be easily operated, to require minimal attention and simple daily maintenance during operation.


Journal of Automatic Chemistry, Vol. 17, No. 6 (November–December 1995), pp. 213–218

### Evaluation of a random access analyser: BM/Hitachi 911

**Taweesook Kanluan, Surapon Tangvarasittichai and Orathai Tangvarasittichai**
*Department of Clinical Chemistry, Faculty of Medical Technology, Mahidol University, Siriraj Hospital, Bangkok 10700, Thailand*

*The performance of Boehringer Mannheim's BM/Hitachi 911 was evaluated for three months. The mean coefficient of variation (CV) of the within-run and between-run imprecision of the 16 analytes were less than 1·16% (range 0·47–2·38%) and 1·35% (range 0·62–2·93%), respectively. A linearity study for the various assays covered clinically important levels. No relevant drift was observed during an eight-hour assay nor was any sample-related carry-over detected. In all cases, the regression analyses (slopes) of the results obtained from BM/Hitachi 911 and 717 were between the extreme values of 0·94 and 1·05. During the three months of operation, no major problem was encountered. The BM/Hitachi 911 was found to be easily operated, to require minimal attention and simple daily maintenance during operation.*

### Introduction

Objective analytical performance evaluations are important to clinical laboratories when looking at the selection of new instruments [1–4]. The BM/Hitachi 911 is a very recent selective access analyser from Boehringer Mannheim GmbH. The photometric unit of the 911 allows the grating spectrophotometer unit to be used in monochromatic or bichromatic mode at 12 fixed wavelengths. The cycle time per test is 20 seconds and the throughput is 360 photometric tests an hour; throughput can be increased if the ISE unit is used.

The two-reagent disk units contain all the test materials necessary for 52 different analytical procedures with 800 routine samples, 800 rerun samples and 200 stat samples. The sample volumes range from 3–50 µl, in 1 µl stepwise increments. The reagent probe is capable of delivering maximum reagent volume of 350 µl and minimum volume of 250 µl. The analyser tested was equipped with an optical bar-code reader for a primary tube in sample disks and in both reagent disks and an RS-232 interface allows a bidirectional link to a host computer.

The study reported in this paper evaluated the performance of BM/Hitachi 911 with 16 analytes (see table 1). Within-run and between-run imprecisions, linearity, analyte drift, sample carry-over and correlation are reported.

### Materials and methods

### *Instruments*

A BM/Hitachi 717 (Boehringer Mannheim GmbH) was used for comparison with the BM/Hitachi 911 (Boehringer Mannheim GmbH)

### *Materials:*

All reagents and calibrators, unless otherwise stated, were from Boehringer Mannheim GmbH and were prepared as described in the manufacturer's literature.

*Table 1. Test, method and assay condition used on the BM/Hitachi 717 and BM/Hitachi 911.*

| Analyte | Method | Wavelength (nm) | | Volume used (µl) | |
|---|---|---|---|---|---|
| | | Main | Sub | Sample | Total reagent |
| Glucose | Glucose oxidase | 505 | 700 | 3 | 253 |
| BUN | Urease (UV) | 340 | 415 | 4 | 404 |
| Creatinine | Jaffé reaction | 505 | 570 | 15 | 327 |
| Uric acid | Uricase-POP-PAP | 505 | 700 | 7 | 307 |
| Cholesterol | Cholesterol oxidase-PAP | 505 | 700 | 3 | 303 |
| Triglyceride | GPO-PAP | 505 | 700 | 3 | 303 |
| Total protein | Biuret | 546 | 700 | 7 | 507 |
| Albumin | BCG | 600 | 700 | 3 | 353 |
| Total bilirubin | Dichlorophenyl (DPD) method | 570 | 700 | 7 | 307 |
| AST | SCE method | 340 | 376 | 15 | 290 |
| ALT | SCE method | 340 | 376 | 15 | 290 |
| Alk. phosphatase | PNP | 415 | 700 | 11 | 311 |
| Total calcium | O-cresolphthalein complexone | 546 | 700 | 10 | 360 |
| Phosphate | Ammonium molybdate (UV) | 340 | 376 | 5 | 365 |
| LDH | Pyruvate-lactate | 340 | 376 | 7 | 307 |
| CK | Optimized standard method (UV) | 340 | 415 | 7 | 307 |

0142-0453/95 $10.00 © 1995 Taylor & Francis Ltd.

*Specimens:*

One hundred serum samples, ranging from normal to pathological values, were used in the study. Each serum was divided equally and assayed either in the BM/Hitachi 911 or 717. The comparative study was obtained by regression analysis of the values of each serum for 16 analytes determined using minimized sum of squares. The linearity study was carried out using high level concentration specimens.

*Control sera:*

The control sera used were:

(1) Boehringer Precinorm lot 175303 (Germany).

(2) Corning lot 020002, 020103, 025103 and 037101 (USA). In method calibration, the same calibrator (lot 759350) was used on both of the BM/Hitachi 911 and 717.

*Table 2. Within-run and between-run imprecision of 16 analytes at three concentrations (N = 20).*

| Analyte (unit) | Within-run Mean | Within-run SD | Imprecision CV(%) | Between-run Mean | Between-run SD | Imprecision CV(%) |
|---|---|---|---|---|---|---|
| Glucose | 6·52 | 0·05 | 0·63 | 6·54 | 0·04 | 0·68[a] |
| (mmol/l) | 9·72 | 0·11 | 1·09 | 9·74 | 0·11 | 1·12[b] |
| | 17·00 | 0·11 | 0·60 | 17·05 | 0·11 | 0·62[c] |
| BUN | 4·13 | 0·04 | 0·93 | 4·18 | 0·05 | 1·23[a] |
| (mmol/l) | 8·52 | 0·15 | 1·78 | 8·62 | 0·17 | 1·95[b] |
| | 13·04 | 0·18 | 1·42 | 13·13 | 0·20 | 1·56[c] |
| Creatinine | 189·17 | 4·51 | 2·38 | 190·94 | 5·39 | 2·82[a] |
| (μmol/l) | 369·07 | 7·60 | 2·06 | 374·90 | 8·31 | 2·22[b] |
| | 720·46 | 6·36 | 0·88 | 721·32 | 7·16 | 0·99[c] |
| Uric acid | 294·00 | 3·03 | 1·03 | 296·79 | 4·88 | 1·64[a] |
| (μmol/l) | 513·99 | 4·82 | 0·94 | 590·73 | 5·35 | 0·91[b] |
| | 614·83 | 5·53 | 0·90 | 622·26 | 5·71 | 0·92[c] |
| Cholesterol | 3·10 | 0·02 | 0·68 | 3·11 | 0·03 | 0·84[a] |
| (mmol/l) | 3·36 | 0·59 | 1·75 | 3·36 | 0·06 | 1·82[b] |
| | 4·44 | 0·02 | 0·47 | 4·45 | 0·02 | 0·52[c] |
| Triglyceride | 1·32 | 0·01 | 0·76 | 1·32 | 0·01 | 0·76[a] |
| (mmol/l) | 1·60 | 0·01 | 0·63 | 1·61 | 0·01 | 0·62[b] |
| | 1·73 | 0·02 | 1·15 | 1·74 | 0·02 | 1·15[c] |
| Total protein | 50·81 | 0·98 | 1·93 | 51·01 | 1·01 | 1·98[a] |
| (g/l) | 51·00 | 0·31 | 0·61 | 51·50 | 0·40 | 0·77[b] |
| | 61·13 | 0·35 | 0·57 | 62·00 | 0·41 | 0·66[c] |
| Albumin | 30·20 | 0·30 | 0·99 | 31·00 | 0·45 | 1·45[a] |
| (g/l) | 36·10 | 0·51 | 1·41 | 36·40 | 0·56 | 1·54[b] |
| | 36·53 | 0·52 | 1·42 | 36·78 | 0·64 | 1·74[c] |
| Total bilirubin | 35·91 | 0·72 | 2·00 | 37·62 | 0·87 | 2·32[a] |
| (μmol/l) | 72·73 | 1·42 | 1·95 | 73·89 | 1·56 | 2·10[b] |
| | 119·87 | 1·44 | 1·20 | 121·41 | 1·47 | 1·21[c] |
| AST | 50·60 | 0·49 | 0·96 | 51·20 | 0·91 | 1·78[a] |
| (U/l) | 104·92 | 1·89 | 1·80 | 108·12 | 1·98 | 1·83[b] |
| | 205·40 | 1·92 | 0·93 | 206·10 | 1·94 | 0·94[c] |
| ALT | 44·60 | 0·54 | 1·21 | 45·10 | 0·62 | 1·37[a] |
| (U/l) | 88·85 | 1·14 | 1·28 | 89·94 | 1·25 | 1·38[b] |
| | 106·80 | 0·94 | 0·88 | 107·20 | 1·21 | 1·13[c] |
| ALP | 74·31 | 1·03 | 1·38 | 75·24 | 1·13 | 1·49[a] |
| (U/l) | 224·81 | 2·14 | 0·95 | 225·40 | 2·41 | 1·07[b] |
| | 317·46 | 4·07 | 1·28 | 318·42 | 4·10 | 1·29[c] |
| Total calcium | 2·13 | 0·05 | 2·35 | 2·14 | 0·06 | 2·93[a] |
| (mmol/l) | 3·07 | 0·05 | 1·70 | 3·12 | 0·07 | 2·40[b] |
| | 3·36 | 0·05 | 1·48 | 3·38 | 0·07 | 2·07[c] |
| Phosphate | 1·59 | 0·02 | 1·14 | 1·61 | 0·02 | 1·22[a] |
| (mmol/l) | 2·38 | 0·03 | 1·10 | 2·41 | 0·03 | 1·31[b] |
| | 2·41 | 0·03 | 1·05 | 2·42 | 0·03 | 1·25[c] |
| LDH | 292·10 | 2·16 | 0·74 | 292·50 | 2·46 | 0·84[a] |
| (U/l) | 586·69 | 4·10 | 0·70 | 589·97 | 4·90 | 0·83[b] |
| | 955·20 | 5·10 | 0·53 | 957·10 | 8·10 | 0·85[c] |
| CK | 248·10 | 2·13 | 0·86 | 249·40 | 2·48 | 0·99[a] |
| (U/l) | 439·00 | 3·10 | 0·71 | 441·24 | 3·41 | 0·77[b] |
| | 505·00 | 2·71 | 0·54 | 506·20 | 3·41 | 0·67[c] |

Where: *a* = Boehringer precinorm lot 175303, Germany; *b* = Ciba Corning: lot 020002, USA; *c* = Ciba Corning: lot 037101, USA.

*Table 3. Sample-related carry-over of 16 analytes.*

| Analyte | Unit | Concentration | | % Carry-over, when high as contaminant | %Carry-over, when low as contaminant |
|---|---|---|---|---|---|
| | | High (h) | Low (l) | | |
| Glucose | mmol/l | 16·2 | 4·6 | 0 | −0·34 |
| BUN | mmol/l | 8·9 | 2·3 | 0·71 | −0·57 |
| Creatinine | µmol/l | 725 | 183 | 0 | −1·22 |
| Uric acid | µmol/l | 501 | 291 | 0 | 0 |
| Cholesterol | mmol/l | 4·0 | 3·2 | 0 | −0·81 |
| Triglyceride | mmol/l | 1·8 | 0·9 | −1·2 | −0·61 |
| Total protein | g/l | 61 | 48 | 0 | 0 |
| Albumin | g/l | 40 | 33 | 0 | 0 |
| Total bilirubin | µmol/l | 163 | 10 | 0 | 1·05 |
| AST | U/l | 185 | 32 | 0 | −1·07 |
| ALT | U/l | 92 | 32 | 0 | 1·08 |
| Alk. phosphatase | U/l | 287 | 99 | 0 | −1·36 |
| Total calcium | mmol/l | 3·1 | 2·3 | −1·0 | 0 |
| Phosphate | mmol/l | 2·3 | 0·9 | 0 | −1.39 |
| LDH | U/l | 950 | 316 | 0 | 0·21 |
| CK | U/l | 515 | 142 | 0 | −1·74 |

(3) The reagents for determinations of glucose, AST and ALT were from Wako (Japan) and Ames (Italy), respectively.

*Methods*

The methods and assay conditions used in this study on BM/Hitachi 717 or 911 are summarized in table 1.

### Results

*Imprecision*

Within-run and between-run imprecision was investigated with three different levels of control sera. The within-run imprecision was assayed 20 times in the same batch, and between-run imprecision was tested using the same sera on 20 consecutive batches. Data on the within-run and between-run imprecision are presented in table 2. The percentage of coefficient of variation (% CV) of both assays was less than 3%.

*Accuracy and linearity*

A linearity study was performed using a high concentration control serum diluted with isotonic saline. The diluted sera were assayed in duplicate and the mean values obtained. The difference between calculated target and observed value was used for assessing accuracy. The upper limit of each analyte obtained from the study is shown in figure 1. The upper limits were in close agreement with the expected ranges claimed by the manufacturer.

*Drift*

The drift of 16 analytes was assayed using two control sera analysed at hourly intervals for eight hours. The value determined at zero hours were performed in triplicate and the subsequent determinations were performed once. The pooled sera were aliquoted in tightly closed vials and kept in a refrigerator. Prior to the assay, the aliquot was transferred to a sample cup and left at room temperature for 10 min. None of the analytes showed a deviation more than 5% (see figure 2(*a*) and 2(*b*)).

*Sample carry-over*

Carry-over caused by a sample probe was assayed using Bennet's model (6). The assay was performed in three successive sample portions: high concentration ($h_1 \ldots h_3$), low concentration ($l_1 \ldots l_3$) and same high concentration ($h_1 \ldots h_3$). All samples were assayed in triplicate and the percentage carry-over was calculated as follow:

$$\text{High samples} = \frac{h_1 - h_2}{h_2} \times 100$$

or

$$\text{Low samples} = \frac{l_1 - l_2}{l_2} \times 100$$

Data presented in table 3 show that there was no appreciable carry-over in any analytes. The overall percentage carry-over was less than 2%.

*Correlation*

One hundred samples from normal to pathological levels were divided in half and assayed simultaneously in the BM/Hitachi 911 or 717. Table 4 presents the regression analysis of 16 analytes. The extreme slope values obtained were 0·91 and 1·09, and those for intercepts were −8·22 and 0·61, respectively. This finding suggested that the two instruments performed similarly.

### Discussion

Total analytical imprecision is the summation of the variances arising from both chemical and instrumental factors [1, 7]. In this study, the CVs of between-run imprecision in three control sera were acceptable (<3%).

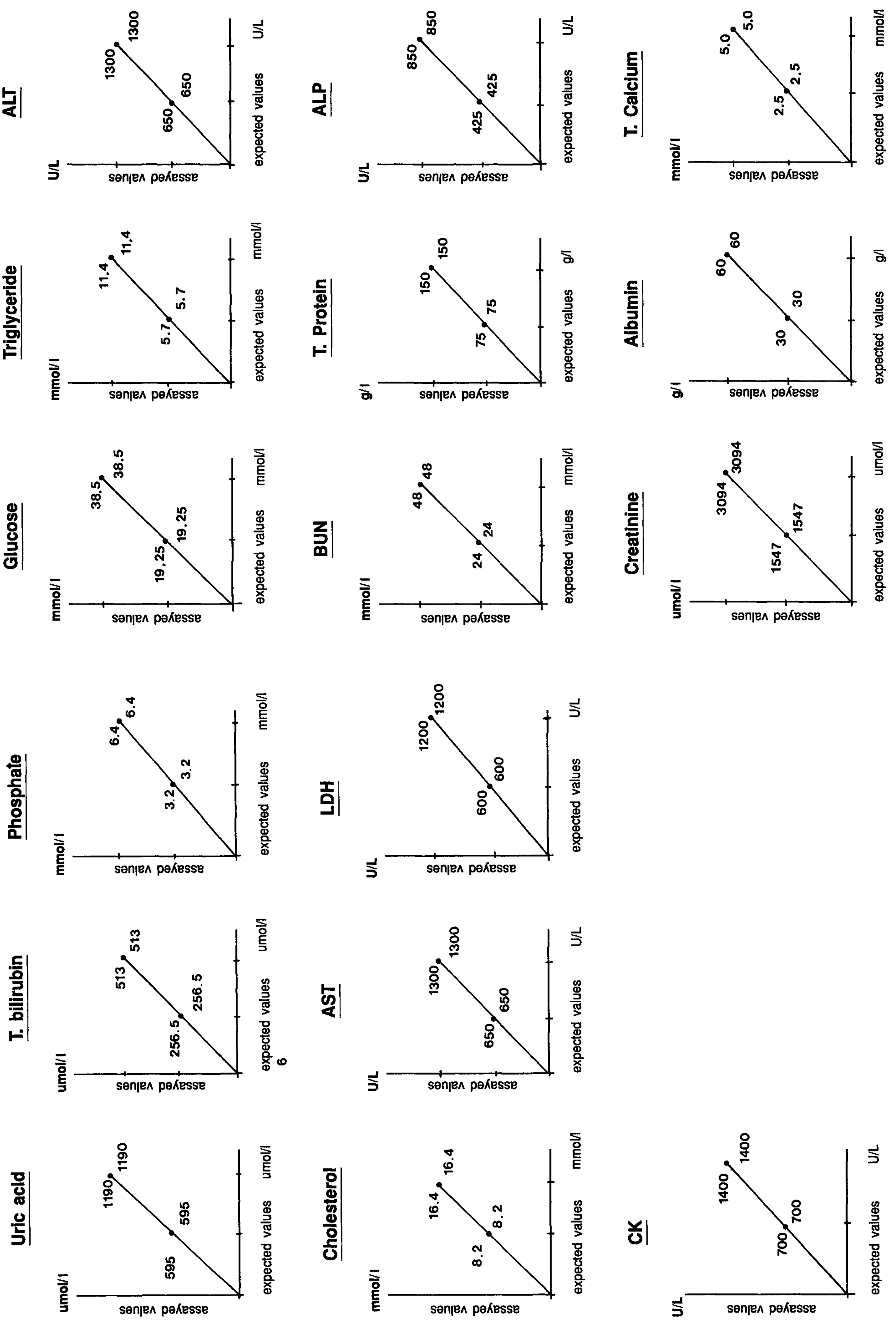

*Figure 1. Linearity of assays performed by the BM/Hitachi 911.*

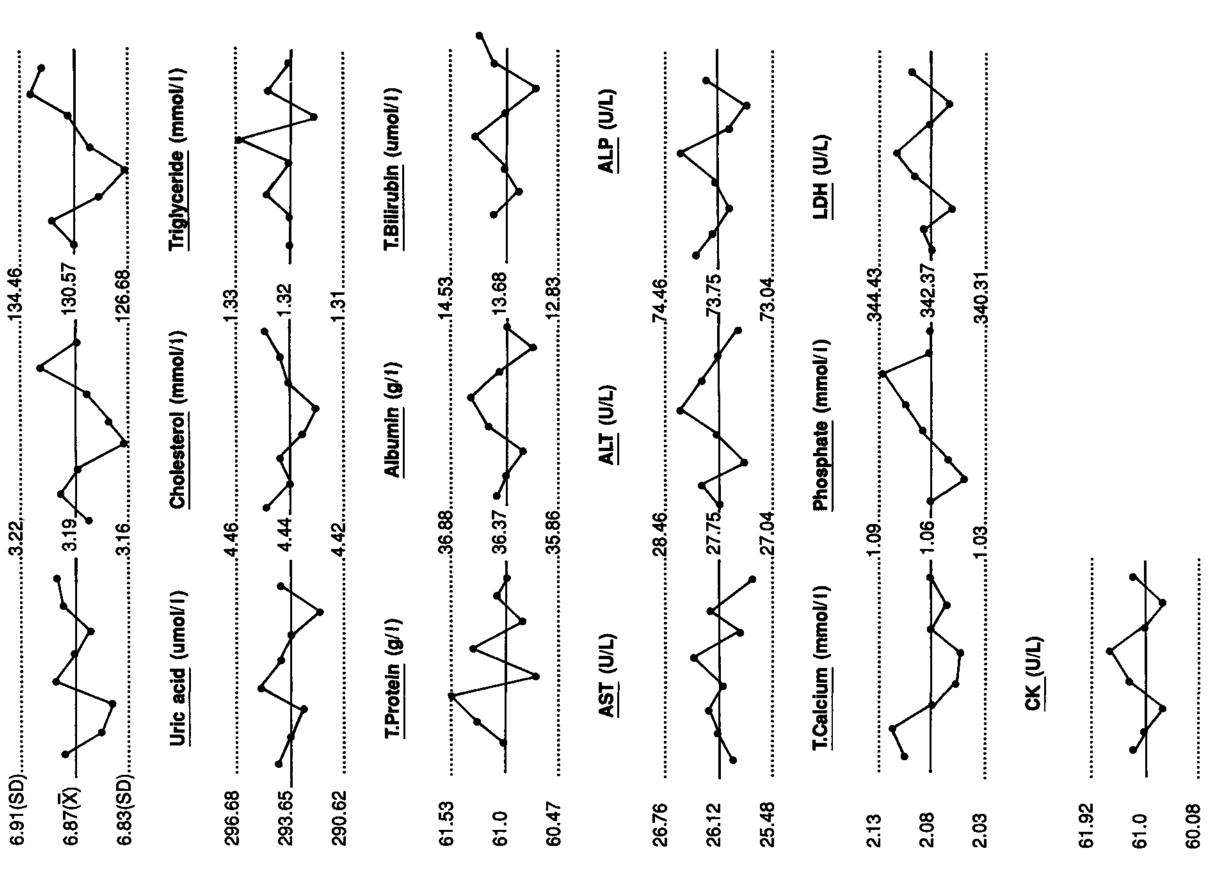

*Figure 2(a). Drift of 16 analytes obtained from control 1 sera during an eight-hour experiment.*

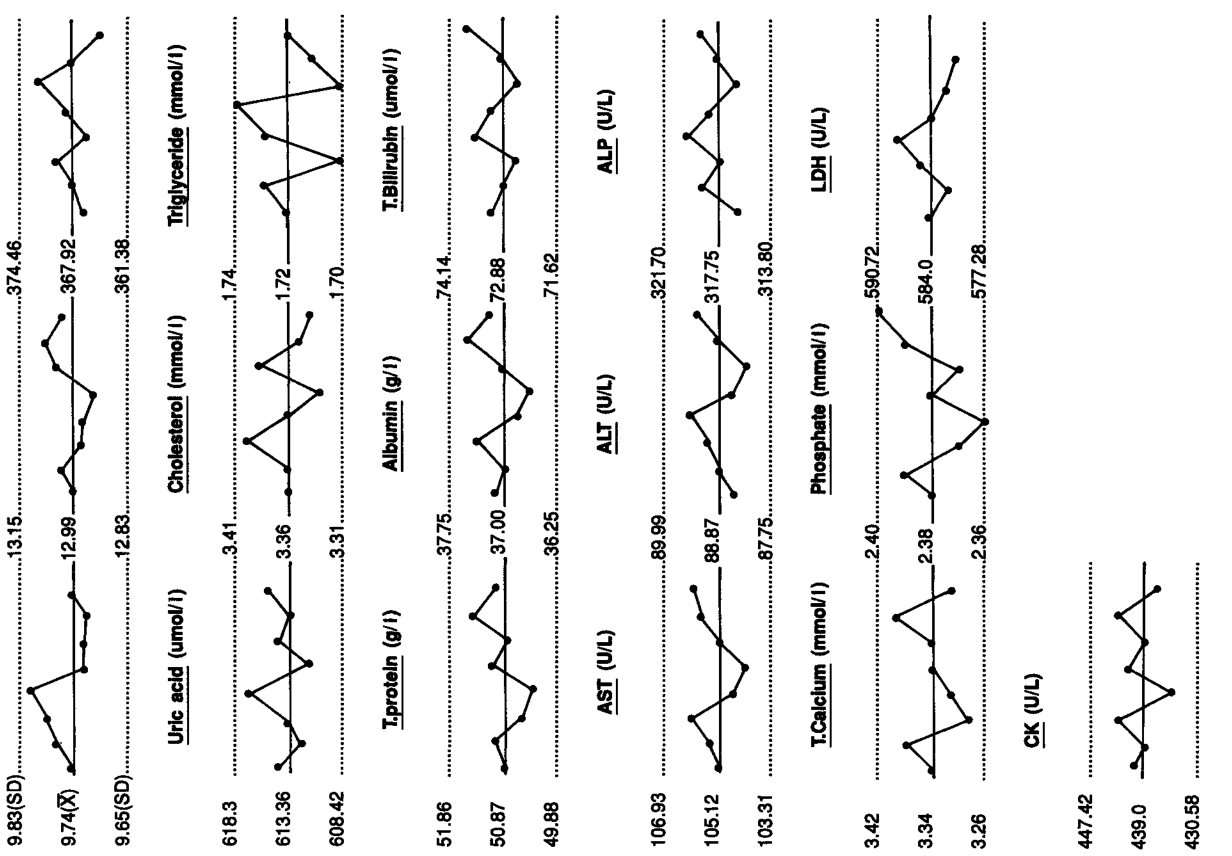

*Figure 2(b). Drift of 16 analytes obtained from control 2 sera during an eight-hour experiment.*

*Table 4. Regression analysis of 16 analytes on BM/Hitachi 717(x) and 911(y), where N = 100.*

| Analyte | Unit | Regression analysis (Y = *bx* + *a*) *b* | *r* | *a* |
|---|---|---|---|---|
| Glucose | mmol/l | 0·96 | 0·99 | −0·007 |
| BUN | mmol/l | 1·05 | 0·98 | −0·023 |
| Creatinine | μmol/l | 1·09 | 0·99 | −8·22 |
| Uric acid | μmol/l | 0·99 | 0·99 | −7·77 |
| Cholesterol | mmol/l | 0·94 | 0·98 | 0·003 |
| Triglyceride | mmol/l | 0·97 | 0·99 | 0·0002 |
| Total protein | g/l | 1·01 | 0·99 | 0·47 |
| Albumin | g/l | 0·99 | 0·99 | 0·10 |
| Total bilirubin | μmol/l | 0·91 | 0·99 | −0·11 |
| AST | U/l | 1·05 | 0·99 | 0·19 |
| ALT | U/l | 1·01 | 0·99 | −0·20 |
| Alk. phosphatase | U/l | 0·98 | 0·99 | −2·83 |
| Total calcium | mmol/l | 0·97 | 0·98 | 0·030 |
| Phosphate | mmol/l | 0·96 | 0·99 | −0·006 |
| LDH | U/l | 1·01 | 0·99 | 0·61 |
| CK | U/l | 0·99 | 0·99 | 0·48 |

According to the quality specification for between-run analytical imprecision proposed by a Working Group of EGE-Lab [8], it was shown that the analytical system achieved these specifications in almost all cases (table 5). This finding reflected the good quality spectrophotometer and pipetting systems. However, the mean values for each analyte in the investigation of imprecision were consistently slightly higher between run compared with within run. This could be due to a slight change in the biological matrix during the storage of control serum. Photometric linearity was adequate in all tests (see figure 1) with no drift detected in any various analytes during an eight-hour assay. There is good correlation ($r$ = 0·97–0·99) between the results obtained from BM/Hitachi 911 and 717. There were no problems during the installation of BM/Hitachi 911; and there were no instrument failures during the evaluation study. Laboratory staff learnt to operate and maintain the equipment within three days. The operator's manual and guidelines for trouble-shooting are easily understood.

In conclusion, the BM/Hitachi 911 fulfilled the acceptance criteria for analytical performance. This instrument is a flexible, convenient and easy-to-use analyser for either batch or random access work. Its design and operational simplicity provides reliable analytical data. The BM/Hitachi 911 is well-suited to routine operation and emergency analyses for small and medium-sized laboratories, and as a back-up system for large laboratories.

*Table 5. Comparison of between-run imprecision proposed by the Working Group of EGE-Lab and BM/Hitachi 911.*

| Analyte | Working Group* | BH/Hitachi 911 % CV | (Mean) |
|---|---|---|---|
| Glucose | 2·2 | 1·12 | (9·74 mmol/l) |
| BUN | 6·3 | 1·95 | (8·62 mmol/l) |
| Creatinine | 2·2 | 2·82 | (190·9 μmol/l) |
| Uric acid | 4·2 | 1·64 | (296·8 μmol/l) |
| Cholesterol | 2·7 | 1·82 | (3·36 mmol/l) |
| Triglyceride | 11·5 | 1·21 | (1·74 mmol/l) |
| Total protein | 1·4 | 1·98 | (51·0 g/l) |
| Albumin | 1·4 [1·8] | 1·74 | (36·8 g/l) |
| Total bilirubin | 11·3 | 2·32 | (37·6 μmol/l) |
| AST | 7·2 | 1·83 | (108·1 U/l) |
| ALT | 13·6 | 1·38 | (89·9 U/l) |
| Total calcium | 0·9 [1·5] | 2·93 | (2·14 mmol/l) |
| Phosphate | 4·0 | 1·31 | (2·41 mmol/l) |
| LDH | 3·9 | 0·85 | (957·1 U/l) |
| CK | 20·7 | 0·99 | (249·4 U/l) |

Where * Proposed quality specification of between-run imprecision (% CV) by a Working Group of EGE-Lab [8].
[ ] interim quality specifications proposed by the Working Group.

### Acknowledgements

The authors wish to express their sincere thanks to Dr Verawan Veinravi for her constructive criticism and reading the paper and to Nida Dakrabutr for her skillful preparation of this manuscript.

### References

1. Broughton, P. M. G., Gowenlock, A. H., McCormack, J. J. and Neill, D. W., *Annals of Clinical Biochemistry*, **11** (1974), 207.
2. Fraser, C. G. and Singer, R., *Clinical Chemistry*, **31** (1985), 667.
3. Kanluan, T., Intaramanee, S. and Tangvorasittichai, S. *et al.*, *Journal of Automatic Chemistry*, **13** (1991), 97.
4. Bonini, P., Coriotti, F. and Frangini, C., *Journal of Automatic Chemistry*, **10** (1988), 167.
5. Okuda, K., *Journal of Clinical Chemistry and Clinical Biochemistry*, **18** (1980), 947.
6. Bennet, A., Gartelmann, D., Mason, J. I., and Owen, J. A., *Clinica Chimica Acta*, **29** (1970), 161.
7. Buttner, J., Borth, R., Boutwell, J. H., Broughton, P. M. G. and Bowyer, R. C., *Journal of Clinical Chemistry and Clinical Biochemistry*, **18** (1978), 69.
8. Fraser, C. G., Hyltoft, Peterson, P., Ricos, C. and Hacokel, R., *Journal of Clinical Chemistry and Clinical Biochemistry*, **30** (1992) 311.

